# Oral Health-Related Quality of Life After Dental Rehabilitation Under General Anesthesia in Children With Early Childhood Caries and Special Health Care Needs

**DOI:** 10.7759/cureus.82272

**Published:** 2025-04-14

**Authors:** Malini Venugopal, Vineetha Karuveettil, Parvathy Kumaran, Krupa R Robert, Arun M Xavier, Vennila Chandran, Nishna Thankappan

**Affiliations:** 1 Pediatric and Preventive Dentistry, Amrita School of Dentistry, Ernakulam, IND; 2 Public Health Dentistry, Amrita School of Dentistry, Ernakulam, IND

**Keywords:** dental rehabilitation, early childhood caries, general anesthesia, oral health-related quality of life, special care dentistry

## Abstract

Objective: Early childhood caries (ECC) has emerged as a prevalent global epidemic. Furthermore, we recognize that children with special health care needs (CSHCN) have a higher prevalence of diseases like ECC. Dental rehabilitation under general anesthesia (DRGA) is an efficient, safe, high-quality treatment modality that can be completed in one appointment, requiring little cooperation from children, and the child does not have any bad memories of treatments. The study aims to examine the impact of comprehensive dental treatment under general anesthesia on the oral health-related quality of life (OHRQoL) of children with ECC, as well as the quality of life of their caregivers.

Methodology: This is a cross-sectional study using the survey method. The study consists of a 13-item questionnaire. The questionnaire is called the Early Childhood Oral Health Impact Scale (ECOHIS). This questionnaire was filled out before DRGA, two weeks and one year after the treatment was done under general anesthesia.

Results: The study population included healthy (7) and special needs children (9). The results indicated that both normal and special children in the present study population had higher total ECOHIS scores. The child impact score was higher and statistically significant among special children (23.00 ± 6.32) in comparison to normal children, indicating poor quality of life. When comparing pre- and post-intervention, there was a significant improvement in ECOHIS scores for normal and special children.

Conclusion: The DRGA significantly improved OHRQoL in ECC children. In CSHCN, the improvement was even greater.

## Introduction

Early childhood caries (ECC) is one of the most challenging oral diseases of childhood, which may begin as soon as a tooth erupts into the oral cavity. If not treated at the appropriate time, ECC can result in a debilitating health condition due to poor nourishment, which will also have an effect on a child’s speech development and communication, as well as interpersonal skills [1]. These consequences may hinder the child’s normal growth and development and affect daily life activities and psychological well-being, leading to a poor quality of life for the affected child and their parents [2]. Children with special health care needs (CSHCN) generally have a higher prevalence of caries than the general population, and providing dental treatment for them can be an added challenge [3]. ECC presents a greater challenge when it comes to providing treatment, as one vital part of managing a child is how the child embraces the required treatment in the dental clinical setup without apprehension or thinking of dental visits as a terror. Despite the abundance of non-pharmacological techniques, the pediatric dentist encounters obstacles in the dental chair due to factors such as the child's young age, complex medical, physical, or mental conditions, or the requirement for extensive emergency care. Therefore, moving to pharmacological methods to achieve a guarded atmosphere for pediatric patients should be considered [4]. Compared to the chairside approach for pediatric dental treatment, dental rehabilitation under general anesthesia (DRGA) for children can be an effective alternative to provide complicated treatment procedures with superior quality and safety. The benefits include completing all necessary treatments in a single appointment and preventing the child from developing apprehension toward dental treatment [5].

When assessing the impact of dental procedures, their influence on the quality of life is just as significant as the clinical outcome. Patients’ satisfaction, subjective emotional experience, and overall functioning are as important as the treatment outcomes [6,7]. An instrument that measures oral health-related quality of life (OHRQoL) can assess the effect of chronic oral diseases on a child’s quality of life [2]. The Early Childhood Oral Health Impact Scale (ECOHIS), a short, valid, and reliable tool, evaluates the impact of oral health problems and encompasses functional restraints, dental problems, and social and emotional contentment [8].

A 2015 study in Kerala, India, revealed an alarmingly high 54% prevalence of ECC [9]. CSHCN also generally have an increased prevalence of dental caries than the general population, and hence it is an added challenge to both caregivers and pediatric dentists [3]. Studies by Farsi et al. and Baens-Ferrer et al. found that DRGA markedly improves OHRQoL in children less than six years old, and the improvement is greater in CSHCN [8,10].

This study is using the Malayalam version of ECOHIS, which has already been validated and used in a study by Bhat and Sivaram in 2015 [11]. Kerala has used the Malayalam version of ECOHIS but has not used it to analyze the impact of DRGA. Only a limited number of studies, exclusively in Western and Middle Eastern countries, have utilized the ECOHIS questionnaire. Owing to cultural and regional differences, there could be a difference in the observations in our population. The aim of the study is to determine the OHRQoL in children, including those CSHCN with ECC after DRGA. It evaluates pre- and post-treatment OHRQoL as well as the quality of life of their caregivers.

## Materials and methods

Study design

A comparative observational longitudinal study (pre-post) on OHRQoL among children who underwent treatment under general anesthesia (GA) at Amrita Institute of Medical Sciences, Kochi, in Kerala, was carried out during the period from December 2020 to December 2021. Comparisons were made within the same group before and after treatment, as well as between healthy children and CSHCN. A one-year follow-up until December 2022 was carried out. Approval was obtained from the Institutional Review Board of Amrita Institute of Medical Sciences (IRB-AIMS-2020-119). The study was explained to the participants, and informed consent was obtained from all the participants before the study.

Sample size

The sample size was estimated based on a previous study (Farsi et al., 2018) [8] that compared ECOHIS scores between healthy children and CSHCN. The mean (SD) ECOHIS scores were 19.9 (10.3) for healthy children and 25.9 (11.3) for CSHCN. Considering a significance level of 5% (α = 0.05) and a power of 80% (1-β = 0.80), the minimum calculated sample size was 5 for healthy children and 4 for CSHCN to detect a significant difference. To account for potential non-responses, we included seven healthy children and six CSHCN. Given the specific nature of the population (children requiring GA for dental rehabilitation), this pilot study aimed to generate preliminary data for future larger studies.

Study participants

Participants were recruited through consecutive sampling from a tertiary care hospital. A total of seven healthy children and nine CSHCN were recruited for the study. The parents of children (including CSHCN) visiting the outpatient department of a tertiary health care hospital with ECC who require DRGA were included in the study. The study excluded parents who did not want to participate and had children older than six years. In the healthy children group, children with any systemic conditions or special health care needs were excluded. Parents who satisfied the inclusion criteria were asked to self-complete the ECOHIS before DRGA.

Questionnaire

The ECOHIS validated questionnaire by Bhat and Sivaram [11], which consists of 13 items in both English and Malayalam (local language), was used in this study. The questionnaire, called the ECOHIS, includes both the Child Impact Scale (CIS) and the Family Impact Scale (FIS), as well as a global question to help assess a child's overall health. This questionnaire was filled out before the treatment, two weeks after the treatment, and under general anesthesia while the child visited for the review. Oral hygiene instructions were given to all the participants and caregivers. A phone call was made to parents who could not attend the following appointment to receive their responses. After one year, the questionnaires were mailed, and parents were contacted over the phone to complete the ECOHIS questionnaire again. The higher the ECOHIS, the poorer the quality of life. Follow-up questionnaires were completed by the same parent who filled out the baseline.

Statistical analysis

The data analysis was done using IBM SPSS Statistics for Windows, Version 20 (Released 2011; IBM Corp., Armonk, New York, United States). The data was analyzed for normality of distribution using the Shapiro-Wilk test. ECOHIS questionnaire results were compared between normal and special children preoperatively, postoperatively, and one year after GA using an independent t-test. We compared pre-, post-, and one-year follow-up within each group using a paired t-test. A p-value less than 0.05 (p < 0.05) was considered statistically significant.

## Results

A total of seven healthy children and nine CSHCN visiting a tertiary health care center in Kerala were included in the study. The data was summarized, tabulated, and expressed as mean and standard deviation. Baseline and follow-up questionnaires were completed by the same caretaker. The caretaker personally filled out the one-month follow-up questionnaires during their follow-up appointment. The questionnaires were mailed to them after one year.

Baseline ECOHIS scores

Total ECOHIS before dental rehabilitation was noted to be higher among special children (37.86 ± 10.27) than healthy (37.67 ± 10.07), but the difference was not statistically significant (p > 0.05, t-test). This indicates ECOHIS scores were comparable between healthy children and special children. However, the subcategory of the CIS showed that special children had a poor quality of life (23.00 ± 6.32) in comparison to healthy children (22.86 ± 7.60) and was statistically significant (p < 0.05, t-test) (Table 1).

**Table 1:** Comparison of Early Childhood Oral Health Impact Scale scores before dental rehabilitation under general anesthesia The Total Early Childhood Oral Health Impact Scale was noted to be higher among special children (37.86 ± 10.27) before dental rehabilitation, but the difference was not statistically significant (p < 0.05). This indicates Early Childhood Oral Health Impact Scale scores were comparable between normal and special children, except for the sub-category of Child Impact Scale, where special children had a poor quality of life (23.00 ± 6.32) in comparison, and it was statistically significant.

Early Childhood Oral Health Impact Scale	Healthy children (N = 7)	Special children (N = 9)	Mean difference	p-value	Confidence intervals
Child Impact Scale	22.86 ± 7.60	23.00 ± 6.32	-0.142	0.989	(-7.60, 7.32)
Child symptoms	3.43 ± 0.79	3.67 ± 1.32	-0.238	0.678	(-1.45, 0.98)
Child function	9.43 ± 2.64	9.67 ± 2.87	-0.238	0.926	(-3.24, 2.76)
Child psychology	6.29 ± 2.63	5.89 ± 2.15	0.572	0.572	(-2.16, 2.95)
Child self-image and social interaction	3.71 ± 2.93	3.78 ± 1.92	0.625	0.250	(-2.66, 2.54)
Family Impact Scale	11.43 ± 2.57	11.11 ± 3.95	0.317	0.150	(-3.39, 4.02)
Parental distress	5.71 ± 2.75	5.78 ± 2.49	-0.063	0.595	(-2.89, 2.75)
Family function	5.71 ± 2.50	5.33 ± 1.87	0.380	0.685	(-1.96, 2.72)
Total Early Childhood Oral Health Impact Scale score	37.67 ± 10.07	37.86 ± 10.27		0.607	(-10.79, 11.17)
Independent t-test; p < 0.05					

Post-treatment ECOHIS scores

Following DRGA, there was a significant decrease in total ECOHIS scores in both groups, indicating improved OHRQoL. Special children continued to show higher ECOHIS scores (23.86 ± 5.58) than normal children (21.00 ± 7.97), but this difference was not statistically significant (p > 0.05, t-test).

Among the ECOHIS subdomains, child self-image and social interaction were the only domains that did not show a significant change after DRGA (p > 0.05, t-test). The largest reduction in scores was observed in the child symptoms domain (p < 0.001) and the parental distress domain (p < 0.05) (Table 2).

**Table 2:** Comparison of Early Childhood Oral Health Impact Scale scores post dental rehabilitation under general anesthesia The Early Childhood Oral Health Impact Scale was noted to be higher among special children compared to normal children (23.86 ± 5.58) post-dental rehabilitation under general anesthesia, but the difference was not statistically significant (p < 0.05).

Early Childhood Oral Health Impact Scale	Healthy children (N = 7)	Special children (N = 9)	Mean difference	p-value	Confidence intervals
Child Impact Scale	14.86 ± 5.24	13.89 ± 4.46	0.968	0.507	(-4.23, 6.17)
Child symptoms	1.57 ± 0.79	1.55 ± 0.89	0.015	0.963	(-0.89, 0.92)
Child function	6.71 ± 2.50	5.78 ± 2.33	0.936	0.978	(-1.66, 3.53)
Child psychology	2.86 ± 0.90	2.78 ± 1.20	0.079	0.213	(-1.09, 1.25)
Child self-image and social interaction	3.71 ± 2.93	3.78 ± 1.92	-0.063	0.625	(-2.66, 2.54)
Family Impact Scale	7.71 ± 1.70	6.78 ± 2.86	0.937	0.085	(-1.70, 3.57)
Parental distress	3.71 ± 0.95	3.11 ± 1.45	0.603	0.101	(-0.76, 1.97)
Family function	4.00 ± 1.53	3.67±1.66	0.333	0.524	(-1.40, 2.07)
Total Early Childhood Oral Health Impact Scale score	21.00 ± 7.97	23.86±5.58		0.339	(-4.76, 10.47)
Independent t-test; p < 0.05					

One-year follow-up ECOHIS scores

The results of the present study revealed that the effect of DRGA on OHRQoL does not seem to be temporary. The effect of DRGA on OHRQoL was constant throughout the follow-up period. One year after DRGA, the ECOHIS scores continued to decrease in both groups. There was a notable improvement in the areas of child function, self-image, social interaction, and FIS. Child self-image and social interaction were the only domains that did not demonstrate considerable change after DRGA. The highest reduction was noted for child symptoms in the child impact section, and in the family impact section, it was parental distress. The total ECOHIS score was 17.56 ± 4.70 in healthy children and 18.71 ± 1.38 in special children. Although special children continued to show slightly higher scores, the difference remained non-statistically significant (p > 0.05, t-test) (Table 3).

**Table 3:** Comparison of Early Childhood Oral Health Impact Scale scores one year after dental rehabilitation under general anesthesia The Early Childhood Oral Health Impact Scale was noted to be higher among special children compared to normal children (18.71 ± 1.38) one year after dental rehabilitation under general anesthesia, but the difference was not statistically significant (p < 0.05).

Early Childhood Oral Health Impact Scale	Healthy children (N = 7)	Special children (N = 9)	Mean difference	p-value	Confidence intervals
Child Impact Scale	11.29 ± 1.11	10.44 ± 2.00	0.841	0.270	(-0.98, 2.66)
Child symptoms	1.57 ± 0.53	1.44 ± 0.52	0.123	0.124	(-0.45, 0.70)
Child function	5.43 ± 0.53	4.78 ± 1.09	0.651	0.123	(-0.32, 1.62)
Child psychology	2.29 ± 0.49	2.22 ± 0.67	0.634	0.951	(-0.58, 0.70)
Child self-image and social interaction	2.00 ± 0	2.00 ± 0	0	NA	NA
Family Impact Scale	5.71 ± 1.50	5.67 ± 2.18	0.048	0.244	(-2.02, 2,12)
Parental distress	2.71 ± 0.76	2.89 ± 1.17	-0.175	0.125	(-1.27, 0.92)
Family function	3.00 ± 1.00	2.78 ± 1.09	0.222	0.979	(-0.92, 1.36)
Total Early Childhood Oral Health Impact Scale score	17.56 ± 4.70	18.71 ± 1.38			(-2.79, 5.12)
Independent t-test; p < 0.05, NA: not applicable					

Comparison of pre-, post-, and one-year ECOHIS scores

A paired t-test analysis showed a significant improvement in ECOHIS scores after DRGA for both healthy and special children. Among healthy children, the ECOHIS scores significantly decreased from baseline to post-treatment (p = 0.001) and from baseline to the one-year follow-up (p = 0.001). However, the difference between post-treatment and one-year follow-up scores was not statistically significant (p = 0.060). Similarly, among special children, ECOHIS scores significantly decreased from baseline to post-treatment (p = 0.008), from post-treatment to one-year follow-up (p = 0.044), and from baseline to one-year follow-up (p = 0.002), indicating sustained improvement in oral health-related quality of life over time (Tables 4-5).

**Table 4:** Comparison of Early Childhood Oral Health Impact Scale scores pre-treatment, post-treatment, and at one-year follow-up for normal children There was a significant improvement in Early Childhood Oral Health Impact Scale scores after rehabilitation under general anesthesia for the normal child when comparing pre-post intervention and pre-one-year intervention.

	Standard error	Confidence interval	p-value
Pre-post	3.104	(9.50, 23.82)	0.001*
Post-one year	1.573	(-0.18, 7.07)	0.060
Pre-one year	3.011	(13.17, 27.06)	0.001*
*Paired t-test; p < 0.05			

**Table 5:** Comparison of Early Childhood Oral Health Impact Scale scores pre-treatment, post-treatment, and at one-year follow-up for special children There was a significant improvement in Early Childhood Oral Health Impact Scale scores after rehabilitation under general anesthesia for the special child across all time points (pre-post intervention, post-one year intervention, and pre-one year intervention).

	Standard error	Confidence interval	p-value
Pre-post	3.598	(5.19, 22.80)	0.008*
Post-one year	2.017	(0.20, 10.08)	0.044*
Pre-one year	3.814	(9.81, 28.47)	0.002*
*Paired t-test; p < 0.05			

## Discussion

The impact oral health will have on a person’s day-to-day functioning and emotional as well as social well-being determines OHRQoL. ECC is a highly prevalent condition that significantly impacts children's quality of life [12]. The treatment of ECC can become challenging in a conventional setting due to various reasons, like the child's very young age and medical condition, as well as in children with behavioral or intellectual disabilities. In those children, DRGA can be a guarded and effective alternative method for dental care [13]. The study provides information about OHRQoL among children in Kerala before and after DRGA. Although various studies in the literature evaluated the effect of DRGA on OHRQoL in children with ECC [14,15], currently, there have not been relevant studies conducted in the state of Kerala depicting the effect of DRGA on OHRQoL and comparing healthy children with CSHCN.

The result showed OHRQoL of children in need of DRGA was significantly impaired. The results were compared to various studies conducted around the world.

In the present study, the total ECOHIS at baseline was noted to be higher (37.86 ± 10.27) for CSHCN than for healthy children (37.67 ± 10.07). Similarly, the OHRQoL of healthy children in Saudi Arabia was lower (19.9 ± 10.3) when compared to those of CSHCN (25.9 ± 11.3) [14]. The total ECOHIS in this study was 37.86 ± 10.27, which was higher than scores from other studies in Lithuania (17.25 ± 5.6) [2], China (18.5 ± 7.2) [16], Turkey (20.6 ± 8.1) [17], Iran (20.38 ± 5.55) [18], and Australia (27.85 ± 9.55) [19]. At baseline, the present study found that the most affected domains were child function, such as difficulty in having food and loss of school days, and the psychological domain, such as trouble sleeping, irritability, and frustration, with family distress and function following closely behind. Child symptoms, self-image, and social interaction followed later. Similarly, studies by Raghu et al. and Farsi et al. found that the child function domain was the most affected, followed by the child symptom domain [1,5]. However, similar investigations among Chinese and Turkish populations yielded contrary results, with baseline FIS scores being higher than CIS scores [17,20].

The cultural differences in the populations would have contributed to the variation in the results because oral symptoms and problems are not perceived in a similar manner universally, and the degree to which they bother different populations is not standardized. A significant improvement in OHRQoL was observed following DRGA, which was sustained through the follow-up period of one year. This is consistent with several earlier studies conducted globally, showing better OHRQoL after DRGA [2,21,22]. The study by Collado et al. also had similar findings, where the ECOHIS dropped significantly from the day of surgery to one month afterward, and that drop stayed significant three months after GA [23]. A study from Lithuania found that total ECOHIS improved one month after DRGA compared to baseline and stayed low during the six-month dental recall [14]. Yawary et al. found that the mean total ECOHIS improved three months after general anesthesia [19].

Similarly, several studies by Li et al., de Souza et al., Gaynor and Thomson, and Grant et al. showed significant changes in the children's OHRQoL following dental rehabilitation under GA [20,21,24,25]. In the present study, all domains exhibited significant improvement. Child symptoms and psychological domains had the lowest scores. The absence of pain and symptoms would have facilitated proper sleep, thereby reducing the child's irritability. Jankauskiene et al. [2] found that among the Lithuanian children, child symptoms and child psychology domain mean scores decreased the most. Farsi et al. [5] observed a similar trend in the Arabian population with a decrease in the mean scores of child symptom domains.

The current study did not show a significant improvement in child function; a plausible explanation could be the extraction of un-restorable teeth, which could make food consumption difficult. Parents' guilt for neglecting their child's oral health may be the reason for the observed FIS scores, which did not show significant changes when pre-post values were compared. These results are in agreement with those of Farsi et al. [5].

After one year of follow-up, all domains exhibited further lower scores. There was a notable improvement in the areas of child function, self-image, social interaction, and FIS. The results of the present study revealed that the effect of DRGA on OHRQoL does not seem to be temporary. The effect of DRGA on OHRQoL was constant throughout the follow-up period. Farsi et al. [26] reported similar results in their study. The improvement in child function could be attributed to the healing of the extraction site and the children's adaptation to the treatment. The parents' relief from the burden of oral symptoms after dental rehabilitation under GA resulted in improved FIS. Further improved oral hygiene and dietary choices by parents may have contributed to long-term benefits [27,28].

In the current study, ECOHIS was noted to be higher among special children compared to normal children (23.86 ± 5.58) post-dental rehabilitation under GA, but the difference was not statistically significant (p < 0.05). Farsi et al. [5] and Duruk et al. [29] reported similar results, showing a greater decline among healthy children compared to CSHCN; this decline may be due to general health conditions, regular doctor visits, drug use, hospitalization, and other factors that impact overall quality of life. Although DRGA is effective, it does not put an end to the effects of ECC; parental agony as well as functional limitation for the child may persist. These findings may be attributed to multiple dental procedures and extractions in most DRGA patients. If DRGA patients do not closely monitor their dietary habits, they are also at increased risk of developing new and recurrent caries lesions, which may include newly erupted teeth as well [30].

Recommendation

Awareness among health care providers and the general public about the importance of a child’s oral health should be reinforced regularly. Neglecting oral health has a significant effect on the overall well-being of the child. In a country like India, DRGA is not readily accepted by parents as well as dental professionals. Awareness and encouragement to accept DRGA as an alternative safe method of management of ECC, if promoted, can, to a large extent, reduce the burden of ECC, as comprehensive treatment can be provided to a large number of children.

Limitations and future directions

We acknowledge that the small sample size limits the generalizability of our findings. However, due to the specific population studied (children requiring GA for ECC management, including CSHCN), participant recruitment was inherently limited. This study was intended as a pilot exploratory study to gather preliminary data on the impact of ECC and dental rehabilitation on OHRQoL. The findings can serve as a foundation for designing larger, multi-center studies in the future. The other limitation of our study was reliance on phone calls and mailed questionnaires for follow-up, which might have caused response bias. However, follow-up questionnaires were completed by the same parent who filled out the baseline.

## Conclusions

The parents of the children reported a remarkable improvement in the children's OHRQoL at the two-week mark and again at the one-year mark following treatment under GA. The child's symptoms and function showed a notable improvement immediately after the treatment, and the change continued at the one-year follow-up. In CSHCN, the improvement was even greater. The parents of children who underwent DRGA expressed complete satisfaction with this treatment mode, as it provided comprehensive care in a single appointment. DRGA had a positive impact on children's as well as families' quality of life. This study will also help to create awareness among other healthcare providers as well as the general public about the importance of a child’s oral health for overall well-being.
